# A yoga intervention for type 2 diabetes risk reduction: a pilot randomized controlled trial

**DOI:** 10.1186/1472-6882-14-212

**Published:** 2014-07-01

**Authors:** Kelly A McDermott, Mohan Raghavendra Rao, Raghuram Nagarathna, Elizabeth J Murphy, Adam Burke, Ramarao Hongasandra Nagendra, Frederick M Hecht

**Affiliations:** 1 Osher Center for Integrative Medicine, University of California, 1545 Divisadero St., San Francisco, CA 94115, USA; 2 Swami Vivekananda Yoga Anusandhana Samsthana, Bangalore, India; 3 Department of Medicine, Division of Endocrinology and Metabolism, University of California, San Francisco, CA, USA; 4 Institute for Holistic Health Studies, San Francisco State University, San Francisco, CA, USA

**Keywords:** Yoga, Prediabetes, Type 2 diabetes, India, Randomized controlled pilot

## Abstract

**Background:**

Type 2 diabetes is a major health problem in many countries including India. Yoga may be an effective type 2 diabetes prevention strategy in India, particularly given its cultural familiarity.

**Methods:**

This was a parallel, randomized controlled pilot study to collect feasibility and preliminary efficacy data on yoga for diabetes risk factors among people at high risk of diabetes. Primary outcomes included: changes in BMI, waist circumference, fasting blood glucose, postprandial blood glucose, insulin, insulin resistance, blood pressure, and cholesterol. We also looked at measures of psychological well-being including changes in depression, anxiety, positive and negative affect and perceived stress. Forty-one participants with elevated fasting blood glucose in Bangalore, India were randomized to either yoga (n = 21) or a walking control (n = 20). Participants were asked to either attend yoga classes or complete monitored walking 3–6 days per week for eight weeks. Randomization and allocation was performed using computer-generated random numbers and group assignments delivered in sealed, opaque envelopes generated by off-site study staff. Data were analyzed based on intention to treat.

**Results:**

This study was feasible in terms of recruitment, retention and adherence. In addition, yoga participants had significantly greater reductions in weight, waist circumference and BMI versus control (weight −0.8 ± 2.1 vs. 1.4 ± 3.6, p = 0.02; waist circumference −4.2 ± 4.8 vs. 0.7 ± 4.2, p < 0.01; BMI −0.2 ± 0.8 vs. 0.6 ± 1.6, p = 0.05). There were no between group differences in fasting blood glucose, postprandial blood glucose, insulin resistance or any other factors related to diabetes risk or psychological well-being. There were significant reductions in systolic and diastolic blood pressure, total cholesterol, anxiety, depression, negative affect and perceived stress in both the yoga intervention and walking control over the course of the study.

**Conclusion:**

Among Indians with elevated fasting blood glucose, we found that participation in an 8-week yoga intervention was feasible and resulted in greater weight loss and reduction in waist circumference when compared to a walking control. Yoga offers a promising lifestyle intervention for decreasing weight-related type 2 diabetes risk factors and potentially increasing psychological well-being.

**Trial registration:**

ClinicalTrials.gov Identified NCT00090506.

## Background

Type 2 diabetes mellitus (T2DM) is a major global health problem with a prevalence of 366 million in 2011 that is projected to increase by 51%, reaching 552 million by 2030 [1,2]. India follows this global trend with a prevalence of 60 million in 2011 that is projected to increase by 63%, reaching 98 million by 2030 [1]. Factors contributing to the high prevalence of T2DM in India include genetic predisposition exacerbated by environmental factors including increasing prosperity and urbanization resulting in increased abdominal obesity and insulin resistance [3,4].

In India, there is also a high prevalence of prediabetes, with elevated fasting blood glucose (FBG) in the range of 5.6-6.9 mmol/L [3,5]. Individuals with prediabetes are at an increased risk of developing T2DM (FBG ≥7 mmol/L); 5-10% will develop T2DM within one year, 25% within 5 years [6]. Metabolic syndrome is another characterization of high risk individuals with at least three of the following five risk factors: elevated FBG, elevated blood pressure, low high-density lipoproteins (HDL), elevated triglycerides and abdominal obesity [7]. Lower cutpoints for waist circumference plus additional criteria for body mass index (BMI) and truncal subcutaneous fat have been recommended as criteria for metabolic syndrome in the Indian population based on their predisposition for T2DM and cardiovascular disease (CVD) [4,8].

Lifestyle interventions including exercise have been effective in offsetting T2DM complications and the progression from prediabetes or metabolic syndrome to T2DM [6,9-11]. By modifying muscle fibers and enhancing beta cell functions, exercise can optimize insulin sensitivity and improve glucose intolerance [12,13]. Thus, exercise may be particularly effective in earlier disease stages and has been associated with a 30% reduction in metabolic syndrome [14] and a T2DM risk reduction of 63-65% in people with prediabetes [15].

In India there is a rich history of using yoga to manage T2DM [3]. Yoga is a mind/body practice based on traditional Indian philosophy, often incorporating three major components: held or sequences of physical postures, breathing exercises and meditation [16,17]. Yoga’s energy expenditure is similar to other low to moderate exercise [18-20] and a recent review found yoga had beneficial outcomes similar to those of moderate exercise in populations with T2DM [21]. Subsequent to the current pilot study, members of our group completed a trial of n = 277 participants in Bangalore, India with T2DM, comparing a yoga program aimed at T2DM management with an exercise program of comparable intensity. In that study yoga was similar to exercise in terms of reducing FBG, hemoglobin A1c (HbA1c), triglycerides, total cholesterol and very low-density lipoproteins (VLDL) [22]. Evidence suggesting that yoga may also be an effective preventive practice in high risk populations, such as with prediabetes and/or metabolic syndrome, is also accumulating [23]. Investigators recently concluded a large randomized controlled trial of restorative yoga vs. stretching for participants with metabolic syndrome and found that the yoga group had significant reductions in FBG that were sustained at 12 months [24].

Additional benefits of yoga include improved exercise-related self-efficacy [25] quality of life [26] and mood [27,28] all of which are important factors in maintenance of lifestyle behavior change. Yoga also may have a different effect on the sympathetic nervous system (SNS) and hypothalamus-pituitary-adrenal (HPA) axis response to stress compared to other types of exercise. Where other exercise can stimulate the SNS/HPA axis, yoga may be down regulating it, shifting to parasympathetic dominance of the stress response [21,23]. In Nagarathna et al., authors found that yoga was superior to exercise at decreasing the need for oral hypoglycemia medications, decreasing low-density lipoproteins (LDL) and increasing HDL, suggesting a benefit associated with yoga in addition to that of just increasing exercise [22].

The current pilot study compares yoga to a walking control among Indians in Bangalore over an 8-week intervention period. This study is a product of an international collaboration to share areas of expertise between the Swami Vivekananda Yoga Anusandhana Samsthana (SVYASA) in Bangalore, India and the Osher Center for Integrative Medicine (OCIM) at the University of California, San Francisco (UCSF). The pilot study aimed to establish feasibility based on meeting recruitment, retention and adherence goals and to collect preliminary efficacy data on changes in T2DM risk factors and psychological well-being.

## Methods

### Study design and participants

**Figure 1:**
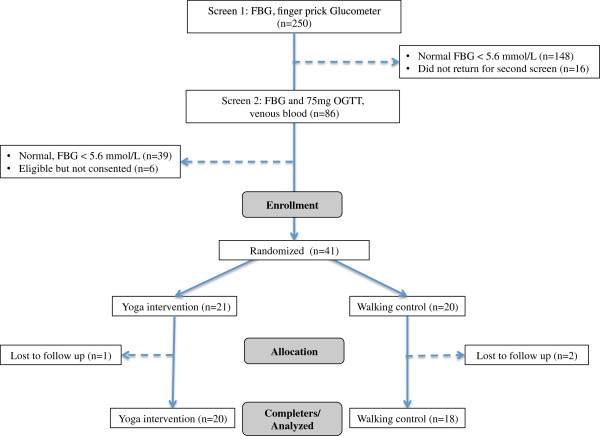
Flow diagram of study enrollment.

This was a 1:1 randomized controlled pilot study conducted in Bangalore, India. The study was reviewed and approved by the research ethics committees at SVYASA and UCSF. Written, informed consent was obtained from all participants. Figure 1 depicts a flow diagram of study screening, enrollment and analysis. Participants were recruited over one week, (October 24, 2004 – November 1, 2004) using advertisements placed in primary care and diabetes clinics, as well as strategic locations throughout the city. Advertisements invited individuals with a first-degree relative with T2DM and interest in participating in a yoga program to contact the study staff to schedule a screening visit. Individuals were asked to come to up to two screening visits. The first visit evaluated FBG using a glucometer and finger stick capillary whole blood. Those with elevated FBG (≥5.6 mmol/L) at the first visit were invited back for a second screening visit. During the second screening visit the glucometer assessment of FBG was repeated to confirm eligibility and an oral glucose tolerance test (OGTT) was performed. The OGTT used a standard 75 gm glucose load dissolved in 300 ml of water ingested over 5 minutes. Glucose was then measured in laboratory assessments of venous blood. OGTT between 7.8-11 mmol/L was in the eligible range. Sixteen percent of participants did not return for the OGTT two-hour blood draw. Because all of these participants had a second FBG measure, the second FBG was used for screening purposes rather than the OGTT.

In addition to having two screening FBG ≥5.6 mmol/L, inclusion criteria for this study also included: 1) a first-degree relative with T2DM; 2) age between 30–65 years; and, 3) willingness to participate in an 8-week yoga program. Exclusion criteria included: 1) an inability to provide informed consent; 2) taking medications associated with insulin resistance (e.g. glucocorticoids, thiazide diuretics, nicotinic acid, β-blockers); 3) prior diagnosis of diabetes or treatment with diabetes medications; 4) active liver disease (with AST or ALT > 3 times the upper limit of normal); 5) anemia with hemoglobin < 85 g/L; 6) pregnant or plans to become pregnant during the course of the study; 7) recent major trauma or surgery that would interfere with participation; and 8) inability to speak either English or Kannada (the local language in Bangalore). Six participants with borderline FBG screening levels were mistakenly enrolled in the study and randomized. These participants are included in the main intention to treat analysis (ITT), but are excluded in the per-protocol analysis. The per-protocol analysis only includes participants meeting the inclusion criteria of having screening FBG ≥5.6 mmol/L.

### Sample size and randomization procedures

Based on this study’s primary feasibility and preliminary efficacy outcomes, sample size was based on resources available. Randomization was performed using computer-generated random numbers and group assignments delivered in sealed, opaque envelopes generated by off-site study staff. Participants were not blinded to their group assignment.

### Intervention

The yoga group attended a day long (eight hour) group counseling session on healthy lifestyle changes with topics on healthy diet, increasing physical activity and smoking cessation. Spouses were invited to attend this group counseling session as well. Participants in the intervention group were then asked to attend at least three, and up to six, yoga classes per week over the eight weeks of the study. Yoga classes were offered on six days of each week. The classes were held in a community hall and taught by two registered Ayurveda medical practitioners with Masters level yoga training from SYVASA. While one instructor demonstrated postures for the class, the other instructor adjusted individual participant’s postures or gave them suitable alternative postures if necessary.

The yoga intervention was designed to manage glucose levels by increasing metabolism, reducing stress and facilitating a positive outlook. In addition, the classes sought to connect participants with a sense of responsibility and control over their health. Each class lasted 75 minutes total and had the following components: diabetes and stress management education (10 minutes); breathing exercises (6 min); loosening exercises (10 min); standing poses (8 min); supine poses (8 min); prone poses (8 min); sitting poses (8 min); relaxation/corpse pose (6 min); chanting exercises and seated meditation (10 min) (see Table 1 for a more detailed account of postures). The total active time in poses was approximately 32 minutes. Evidence suggests that these poses specifically improve muscle metabolism and stress response [29] and the breathing exercises enhance basal metabolic rate [30,31].

**Table 1:** Yoga class material and asana sequencing

***1.***	***Didactics—10 minutes***
	Diabetes causes, complications and lifestyle factors
	Principles, philosophy, and practices related to a yoga based lifestyle program
	Stress response
	Maladaptive behavior and behavior change
	Emotion and coping
***2.***	***Pranayama (Breathing exercises)—6 minutes***
	Hands stretch breathing
	Ankle stretch breathing
	Tiger breathing
	Rabbit breathing
***3.***	***Loosening exercises (any 3)—10 minutes***
	Jogging
	Forward, backward, side bending
	Twisting
	Pavanamuktasana kriya (Supine knees to chest)
	Surya namaskara (3 sun salutations)
***4.***	***Standing asana (any 3)—8 minutes***
	Padahasthasana (Foot hand)
	Ardhachakrasana (Half moon)
	Trikonasana (Triangle)
	Parshvakonasana (Side angle)
	Ardhakati chakrasana (Half wheel)
	Vrikshasana (Tree)
***6.***	***Supine asana—8 minutes***
	Sarvangasana (Shoulder stand)
	Halasana (Plough)
	Matsyasana (Fish)
	Pavanamuktasana (Supine knee chest position)
	Naukasana (Boat)
***7.***	***Prone asana—8 minutes***
	Bhujangasana I, II , III (Cobra)
	Shalabhasana – alternate legs, both (Locust)
	Dhanurasana (Bow)
	Navasana (Boat)
***8.***	***Sitting asana—8 minutes***
	Paschimottanasana (Seated forward bend)
	Vakrasana/ardhamatsyendrasana (Half twist)
	Ustrasana (Camel)
	Sashankasana (Rabbit)
***9.***	***Relaxation Shavasana (Corpse) with guided scan—6 minutes***
***10***	***Chanting *‘OM’ monosyllables, primordial sounds in Indian philosophy**

The yoga intervention was manualized for this study and while yoga instructors received detailed instruction from study staff on how the class was to be conducted, the yoga classes were not observed by study staff. Participants were asked to do a home practice if they were unable to attend at least three classes in a given week and were asked to keep track of each home practice in a daily diary.

### Control

The control group attended the same day long (eight hour) group counseling session on lifestyle changes with their spouses. The control group was asked to do 30 minutes of walking plus breaks for rest on three to six days per week during each of the eight weeks of the study. Including rest breaks, the total time was approximately equivalent to the 75 minute yoga class. They were asked to perform their walking in the park adjacent to the community hall where the daily yoga classes were held. Two study volunteers monitored control group participants’ attendance at daily walks in the park.

### Measures

Primary feasibility outcomes were determined by percent of recruitment, retention and adherence goals met. Study staff blinded to group assignment collected preliminary efficacy data on blood pressure, weight, and waist circumference. Blood pressure was measured as the mean of two measures taken five minutes apart using a mercury sphygmomanometer. Measurement of height (performed only at baseline) used a standard stadiometer and weight was measured and duplicated on a calibrated scale. Waist circumference was measured at the point of minimal circumference at the level of the uppermost lateral border of the right iliac crest at minimal respiration. In addition to the FBG and OGTT measurements, blood was also drawn for a fasting lipid panel and insulin. LDL was calculated directly. Fasting insulin was assessed using a Roche Automated Chemiluminescence analyzer Cobas e411 and Roche Diagnostic’s insulin kits with an intra assay coefficient of variation (CV) of 5.4% and inter assay CV of 6%. We used the calculator available through the Diabetes Trials Unit to apply version 2 of the homeostasis model assessment (HOMA) algorithm to calculate insulin resistance [32].

Secondary outcomes related to psychological well-being included changes in depression, anxiety, positive affect, negative affect, and perceived stress. Depression and anxiety were measured using the Hospital Anxiety and Depression Scale (HADS), a 14-item questionnaire that avoids measuring symptoms such as fatigue that may be influenced by diabetes risk factors [33]. Positive and negative affect were measured using the Positive and Negative Affect Schedule (PANAS), a 20-item questionnaire designed to assess high activation positive affect (e.g. interested, excited, enthusiastic) and high activation negative affect (e.g. upset, irritable, ashamed) [34]. Stress was measured using the Perceived Stress Scale (PSS), a 10-item instrument asking participants how often they had positive and negative experiences related to stress in the prior month [35].

### Data analysis

Within group changes and between group treatment effects associated with participation in the yoga intervention were evaluated using chi-square tests for categorical data and paired t-tests, independent sample t-tests, and Cohen’s d effect sizes (standardized mean difference) for continuous data. Change scores were calculated as post-intervention minus baseline measurements. Given the pilot nature of this study and the relatively small sample size, we did not have adequate power to detect statistically significant differences in the case of small or medium effect sizes. To provide a measure of the observed effect sizes, we report Cohen’s d for primary and secondary outcome measures.

Three participants dropped out for personal reasons unrelated to the study. Based on this and the randomization, we assumed unobserved outcome data was missing at random (MAR) and therefore conducted two primary analyses. First, we excluded dropouts entirely in a complete-case analysis. With outcomes at one follow-up time point, complete case is a valid analysis assuming missing outcomes are MAR [36]. Second, we included dropouts with their last observation carried forward (LOCF) to include all randomized participants for ITT. In this case, the LOCF was the baseline value for the participant who dropped out. Because participants who dropped out did not complete the study, we also assumed that there was no change in their unobserved outcomes. Based on these two assumptions, the LOCF analysis provides a more conservative estimate of the within and between group effects [36].

We replicated the complete case, between group comparison in a per-protocol analysis, excluding the six participants who did not meet the FBG ≥5.6 mmol/L inclusion criteria. We calculated Pearson’s correlation coefficients to further explore relationships between T2DM risk factors and psychological measures. In addition, based on the wide range of ages among enrolled participants (30–65 years) and the concern that a number of T2DM risk factors would vary based on age we conducted a sensitivity analysis to specifically examine whether baseline or change scores differed by age group. We considered p < 0.05 to be statistically significant and all analyses were conducted using Stata Statistical Software: Release 12. College Station, TX: StataCorp LP.

## Results

Recruitment, retention and adherence goals to determine feasibility were all met in this pilot study. We retained 94% of participants, exceeding our goal for feasibility of ≥75% retention. On average, attendance each week for participants in the yoga group did not differ from attendance in the control group (4 ± 0.5 yoga classes per week vs. 3.8 ± 0.5 monitored walks per week, p = 0.13); however, participants in the yoga group had higher overall attendance compared to control (p = 0.02). Participants in the yoga group attended 33.6 ± 4.3 classes over the eight weeks of the study, which is equivalent to 70% of the maximum offered (six classes per week) and 140% of the minimum required (three classes per week). This is compared to attendance in the control group of 30.2 ± 4.3 walking sessions, which is equivalent to 63% of maximum walks offered and 126% of minimum walks required.

The two groups were similar at baseline. In the yoga group, the mean age was 47.0 ± 9.7 and 43% of participants were male compared to the control group where the mean age was 47.2 ± 9.1 and 35% of participants were male (Table 2). In between group comparisons, yoga participants had significantly greater reductions in weight, waist circumference and BMI versus the walking control participants (weight −0.8 ± 2.1 vs. 1.4 ± 3.6, p = 0.02; waist circumference −4.2 ± 4.8 vs. 0.7 ± 4.2, p > 0.01; BMI −0.2 ± 0.8 vs. 0.6 ± 1.6, p = 0.05) (Table 3). There were no significant between group differences in FBG, PPBG, insulin, insulin resistance, blood pressure, cholesterol, or psychological measures of well-being.

**Table 2:** Baseline for Yoga and control groups as mean ± SD unless indicated

	**Yoga**	**Control**
	**(n = 21)**	**(n = 20)**
Age (years)	47.0 ± 9.7	47.2 ± 9.1
Gender (male), n(%)	9 (43)	7 (35)
**Diabetes risk factors**		
Fasting BG (mmol/L)	6.4 ± 0.9	6.7 ± 1.9
Postprandial BG (mmol/L)	8.3 ± 2.6	6.8 ± 1.2
BMI (kg/m2)	28.4 ± 5.3	26.9 ± 3.0
Weight (kg)	69.2 ± 10.8	65.0 ± 7.6
Waist (cm)	92.5 ± 7.3	89.8 ± 7.2
Insulin (pmol/L)	72.3 ± 30.5	57.7 ± 30.1
Insulin resistance	1.4 ± 0.6	1.1 ± 0.6
Systolic (mmHg)	122.9 ± 8.3	127.6 ± 11.4
Diastolic (mmHg)	82.2 ± 9.1	82.3 ± 6.3
LDL (mmol/L)	2.2 ± 0.5	2.2 ± 0.8
Total cholesterol (mmol/L)	5.2 ± 1.2	5.3 ± 0.8
Triglycerides (mmol/L)	1.9 ± 0.8	2.2 ± 1.2
**Psychological factors**		
Anxiety	7.8 ± 5.3	8.3 ± 5.2
Depression	6.0 ± 4.0	6.8 ± 3.8
Positive affect	24.9 ± 7.7	22.4 ± 11.2
Negative affect	15.4 ± 12.7	17.3 ± 11.6
Perceived stress	18.2 ± 5.4	18.0 ± 5.6

BG fasting blood glucose; BMI body mass index; LDL low-density lipoproteins.

**Table 3:** Change scores within yoga and control, and difference between groups with 95% CI and Cohen's d effect size

	**Yoga**	**Control**	**Diff. (Yoga-Ctrl)**	**P diff.**	**Cohen’s d**
	**(n = 20)**	**(n = 18)**	**[95% CI]**		
**Diabetes risk factors**					
Fasting BG (mmol/L)	0	−0.3	0.3 [−0.5 to 1.1]	0.43	0.27
Postprandial BG (mmol/L)^a^	−0.6	0.1	−0.7 [−2.4 to 0.9]	0.38	0.37
BMI (kg/m2)	−0.2	0.6	−0.8 [−1.6 to −0.0]	0.05	0.68
Weight (kg)	−0.8	1.4	−2.3 [−4.1 to −0.4]	0.02	0.80
Waist (cm)	−4.2*	0.7	−4.9 [−7.9 to −1.9]	<0.01	1.11
Systolic (mmHg)	−5.3*	−5.4*	0.1 [−4.8 to 5.1]	0.95	0.02
Diastolic (mmHg)	−5.3*	−3.6*	−1.7 [−7.6 to 4.1]	0.55	0.20
Insulin (pmol/L)	−16.7	4.2	−20.9 [−52.2 to 10.4]	0.19	0.46
Insulin resistance	−0.3	0.1	−0.4 [−1.0 to 0.2]	0.17	0.48
LDL (mmol/L)	−0.1	0.0	−0.2 [−0.6 to 0.3]	0.47	0.24
Total cholesterol (mmol/L)	−0.9*	−0.7*	−0.2 [−0.9 to 0.6]	0.67	0.15
Triglycerides (mmol/L)	−0.1	−0.1	0 [−0.6 to 0.6]	0.95	0.02
**Psychological factors**^**b**^					
Anxiety	−2.1*	−3.4*	1.3 [−0.8 to 3.4]	0.22	0.42
Depression	−2.5*	−3.2*	0.7 [−1.7 to 3.2]	0.55	0.21
Positive affect	4.8*	4.6	0.2 [−4.9 to 5.2]	0.95	0.02
Negative affect	−5.0*	−8.4*	3.4 [−2.3 to 9.0]	0.24	0.41
Perceived stress	−5.7*	−7.9*	2.2 [−1.3 to 5.8]	0.21	0.43

^a^Postprandial BG was calculated based on available data, yoga n = 18 control n = 10.

^b^Three participants were missing data for psychological factors in the control group.

*p < 0.05.

BG blood glucose; BMI body mass index; LDL low density lipoproteins.

**Figure 2:**
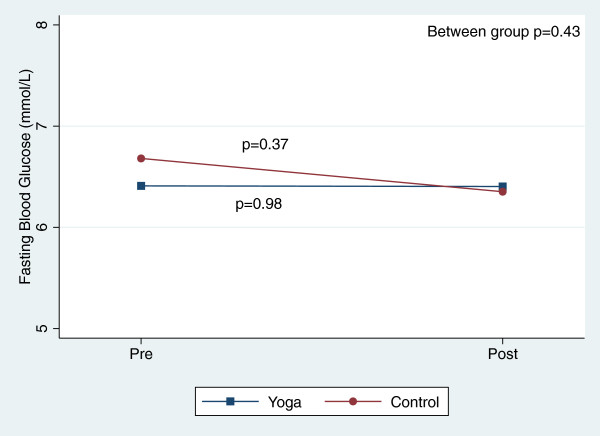
Pre/post changes in fasting blood glucose.

**Figure 3:**
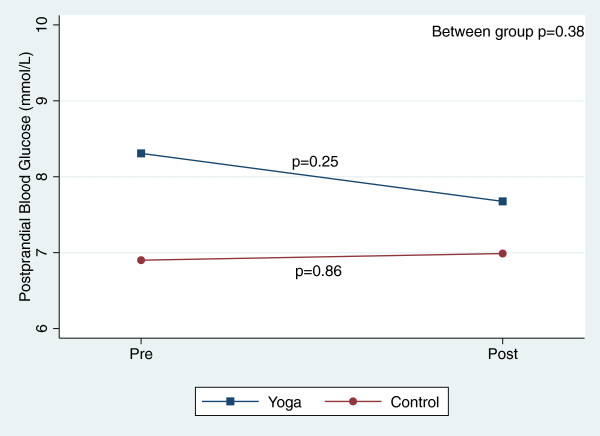
Pre/post changes in postprandial blood glucose.

**Figure 4:**
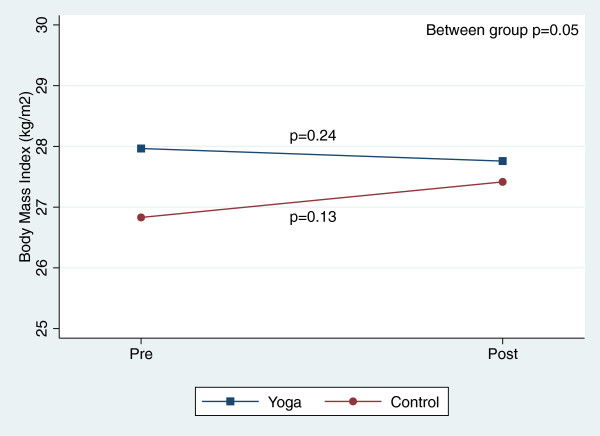
Pre/post changes in body mass index.

**Figure 5:**
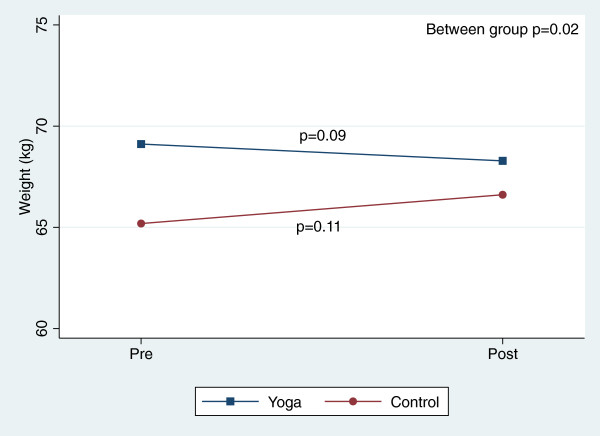
Pre/post changes in weight.

**Figure 6:**
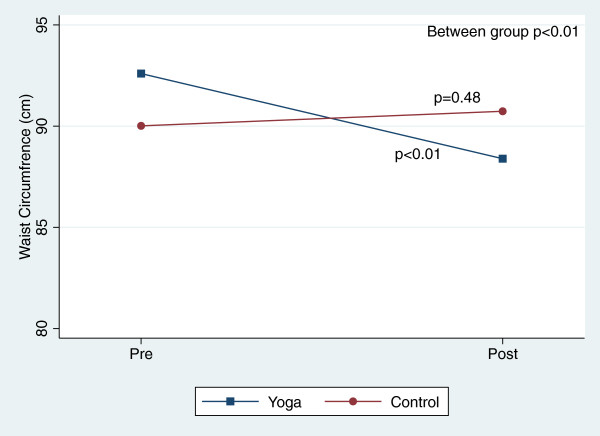
Pre/post changes in waist circumference.

**Figure 7:**
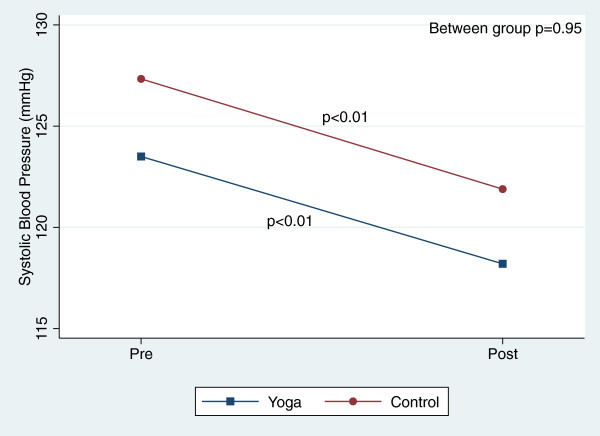
Pre/post changes in serum insulin.

**Figure 8:**
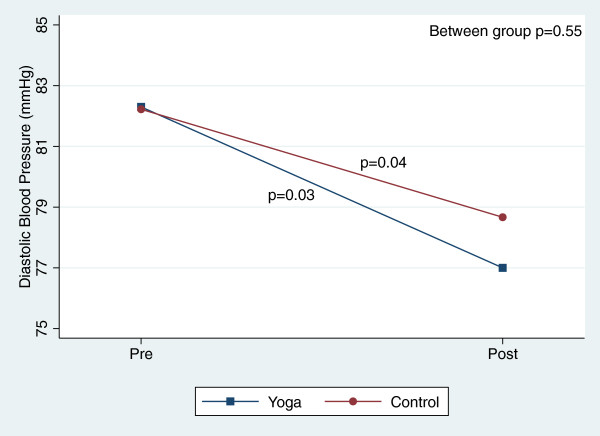
Pre/post changes in insulin resistance.

**Figure 9:**
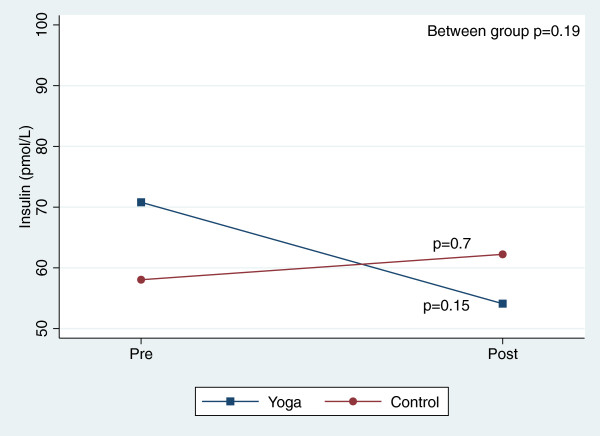
Pre/post changes in systolic blood pressure.

**Figure 10:**
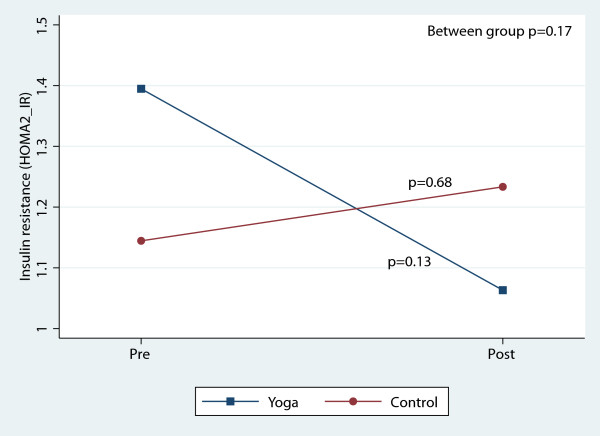
Pre/post changes in diastolic blood pressure.

**Figure 11:**
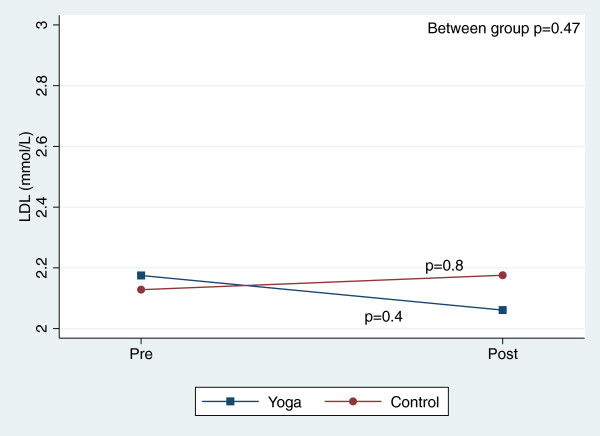
Pre/post changes in low-density lipoproteins.

**Figure 12:**
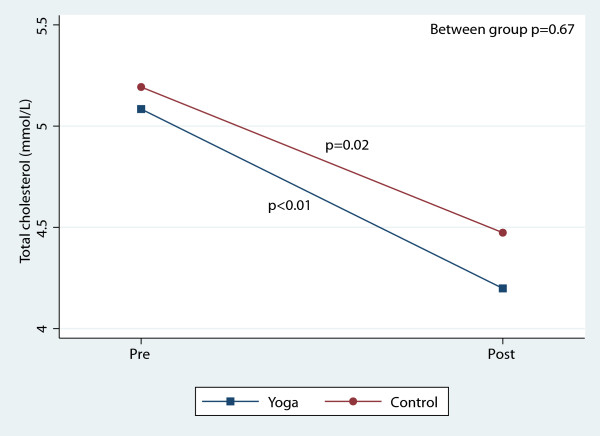
Pre/post changes in total cholesterol.

**Figure 13:**
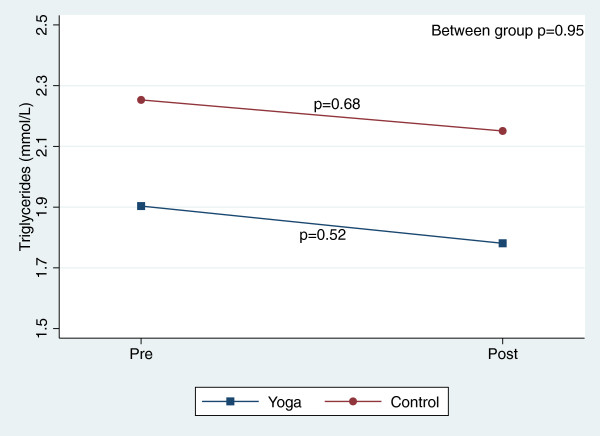
Pre/post changes in triglycerides.

There were, however, significant reductions in blood pressure and total cholesterol within both groups over the course of the 8-week study (Table 3). Figures 2, 3, 4, 5, 6, 7, 8, 9, 10, 11, 12 and 13 illustrate within group changes in T2DM risk factors over time. There were also significant within group improvements in anxiety, depression, negative affect and perceived stress in both groups and a significant within group improvement in positive affect in the yoga group (Table 3). For LOCF analysis, the between group effects were not statistically or substantively different from those reported for the complete case analysis.

In the complete case per-protocol analysis, participants with screening FBG <5.6 mmol/L were excluded (yoga n = 3, control n = 3). The between group reductions in weight, waist circumference and BMI continued to be significant and continued to favor the yoga intervention (Table 4). There were significant within group changes in systolic blood pressure in both groups, diastolic blood pressure in the control group and total cholesterol in the yoga intervention (Table 4). The within group changes in psychological factors in the per-protocol analysis were similar to those found in the main ITT analyses (Table 4).

**Table 4:** **Per-protocol**^**a **^**change scores within yoga and control, and difference between groups with 95% CI and Cohen's d effect size**

	**Yoga**	**Control**	**Diff. (Yoga- Ctrl)**	**P diff.**	**Cohen's D**
	**n = 17**	**n = 15**	**[95% CI]**		
**Diabetes risk factors**					
Fasting BG (mmol/L)	0	−0.5	0.5 [−0.5 to 1.4]	0.32	0.37
Postprandial BG (mmol/L)^b^	−0.4	0.3	−0.7 [−2.8 to 1.4]	0.52	0.32
BMI (kg/m2)	−0.2	0.6	−0.9 [−1.8 to 0.1]	0.07	0.69
Weight (kg)	−1.0	1.5	−2.5 [−4.8 to −0.3]	0.03	0.83
Waist (cm)	−4.9*	0.7	−5.6 [−8.9 to −2.2]	<0.01	1.24
Systolic (mmHg)	−4.4*	−6.5*	2.2 [−3.0 to 7.4]	0.40	0.31
Diastolic (mmHg)	−4.6	−3.9*	−0.7 [−7.1 to 5.6]	0.82	0.08
Insulin (pmol/L)	−14.6	4.1	−18.7 [−54.6 to 17.3]	0.3	0.39
Insulin resistance	−0.3	0.1	−0.4 [−1.1 to 0.3]	0.28	0.40
LDL (mmol/L)	−0.1	0.2	−0.3 [−0.8 to 0.1]	0.17	0.52
Total cholesterol (mmol/L)	−0.9*	−0.7	−0.2 [−1.1 to 0.6]	0.58	0.21
Triglycerides (mmol/L)	−0.2	−0.3	0.1 [−0.6 to 0.7]	0.83	0.08
**Psychological factors**^**c**^					
Anxiety	−1.7*	−3.1*	1.4 [−0.7 to 3.6]	0.19	0.50
Depression	−2.3*	−3.1*	0.8 [−1.9 to 3.4]	0.56	0.22
Positive affect	4.8*	4.7	0.1 [−5.8 to 5.9]	0.99	0.01
Negative affect	−4.5*	−7.6*	3.0 [−2.4 to 8.5]	0.27	0.42
Perceived stress	−6.3*	−8.1*	1.8 [−1.7 to 5.3]	0.31	0.38

^a^Six participants were excluded (yoga n = 3; control n = 3) based on screening FBG <5.6.

^b^Postprandial BG was calculated based on available data, yoga n = 17 control n = 7.

^c^One participant was missing data for psychological factors in the control group.

*p < 0.05.

BG blood glucose; BMI body mass index; LDL low density lipoproteins.

Younger participants had significantly lower LDL and total cholesterol at baseline (30–39 years 1.6 ± 0.5; 40–49 years 2.2 ± 0.6; 50–59 years 2.6 ± 0.6, ≥60 years 2.39 ± 0.4, p = 0.013) but there were no differences in change scores based on age. To explore potential relationships further, we examined correlations between changes in T2DM risk factors and psychological factors within the yoga group. We found significant correlations among changes in negative affect and PPBG (r = 0.49, p = 0.03) and changes in perceived stress and total cholesterol (r = −0.47, p = 0.04). We also saw trends for correlations between changes in depression and PPBG (r = 0.46, p = 0.06); positive affect and insulin (r = 0.40, p = 0.08); perceived stress and BMI (r = 0.42, p = 0.06); perceived stress and weight (r = 0.43, p = 0.06); and positive affect and insulin resistance (r = 0.42, p = 0.07).

## Discussion

Both the yoga intervention and walking control groups in this study exceeded recruitment, retention and adherence goals, meeting feasibility criteria. We found a significant decrease in weight, BMI and waist circumference in the yoga group compared to the walking control, however we did not see a decrease in FBG, PPBG, insulin, insulin resistance, blood pressure, or cholesterol. We did find significant within group decreases in blood pressure and total cholesterol in both yoga and walking control groups as well as within group improvements in measures of psychological well-being.

In the yoga group, positive changes in weight, BMI and waist circumference are similar to other reports in the literature [23,25,37], while the lack of within group change in FBG is contrary to other reports in similar high risk populations [23,24]. In the per-protocol analysis we excluded participants with very low baseline FBG (those in the normal range) and still saw no change in FBG. Our null results were similar to another small study of yoga for high risk individuals (those with a first degree relative with T2DM plus one of the following: impaired FBG, prehypertension, obesity, or abnormal cholesterol) [25] supporting the notion that we may have lacked power to detect small to medium FBG changes. Over time, improvements in weight and waist circumference could yield additional improvements in FBG and related measures.

While both groups had statistically significant within group improvements in measures of psychological well-being, there were no significant differences between groups. The control group likely experienced significant improvement in psychological well-being based on the walking that they were asked to participate in three to six days a week. While we had initially hypothesized that the yoga would improve mood based on a number of factors, the effect of increased walking in the control group appears to have had an equally powerful effect. There was a significant within group increase in positive affect in the yoga group and not in the walking control, however, the between group difference was not significant. In a recent large randomized controlled trial, investigators found that increasing positive affect significantly improved physical activity maintenance at 12 months suggesting a potential behavior change advantage for the yoga group [38].

There are several important limitations in our study. First, several participants did not complete the OGTT, resulting in missing data and limiting our use of OGTT to establish initial eligibility. Second, six participants with borderline FBG did not meet the inclusion criteria of FBG ≥5.6 mmol/L but were mistakenly enrolled and randomized in this study. By including these participants, we are not addressing our original research question whether yoga reduces T2DM risk among participants with FBG ≥ 5.6 mmol/L. To address this, we also included a per-protocol analysis excluding the six participants with FBG <5.6 mmol/L. Third, this study was conducted in India where yoga originated and continues to maintain significant cultural influence. This potentially limits the generalizability of feasibility and adherence findings outside of India. Finally, given the modest sample size in this pilot study, we were not able to detect differences other than large effects.

## Conclusions

In summary, our study has several implications for future investigations of yoga-based interventions for individuals at high risk of T2DM. Our results indicate that yoga is a feasible intervention strategy and may help reduce weight, BMI and waist circumference, three important factors in T2DM risk. We did not find clear evidence of benefit in FBG, PPBG, or insulin resistance however, over time, improvements in weight and waist circumference could yield additional improvements in these parameters. To further test the potential benefits of yoga for T2DM prevention, our results suggest that studies of longer duration and larger sample size are important.

## Abbreviations

BMI: Body mass index; CI: Confidence intervals; FBG: Fasting blood glucose; HADS: Hospital Anxiety and Depression Scale; HOMA: Homeostatic model assessment (version 2); METs: Metabolic equivalent of task; NCCAM: National Center for Complementary and Alternative Medicine; OCIM: Osher Center for Integrative Medicine; OGTT: Oral glucose tolerance test; PANAS: Positive and Negative Affect Schedule; PPBG: Postprandial blood glucose; PSS: Perceived Stress Scale; SVYASA: Swami Vivekananda Yoga Anusandhana Samsthana; T2DM: Type 2 diabetes mellitus; UCSF: University of California, San Francisco.

## Competing interests

The authors declare that they have no competing interests.

## Authors’ contributions

FMH, MRR, RN, EJM, AB, and RHN conceived of the study and participated in design and coordination of preliminary work. MRR, RN and RHN conducted and oversaw staff functions in recruitment, screening and primary data collection in Bangalore, India. KAM, MRR, and FMH analyzed the data and drafted the manuscript. All authors read and approved the manuscript.

## Pre-publication history

The pre-publication history for this paper can be accessed here:

http://www.biomedcentral.com/1472-6882/14/212/prepub
